# Editorial: Insights on fungal diversity of ascomycetes and basidiomycetes: taxonomy and interaction with their host

**DOI:** 10.3389/fmicb.2023.1245204

**Published:** 2023-07-14

**Authors:** Yong-Zhong Lu, Jian-Kui Liu, Jie Chen, Xiang-Yu Zeng, Ji-Chuan Kang

**Affiliations:** ^1^Engineering and Research Center for Southwest Bio-Pharmaceutical Resources of National Education Ministry of China, Guizhou University, Guiyang, Guizhou, China; ^2^School of Pharmaceutical Engineering, Guizhou Institute of Technology, Guiyang, Guizhou, China; ^3^School of Life Science and Technology, University of Electronic Science and Technology of China, Chengdu, China; ^4^Unidad Académica de Biotecnología y Agroindustrial, Universidad Politécnica de Huatusco, Huatusco, Mexico; ^5^Department of Plant Pathology, College of Agriculture, Guizhou University, Guiyang, Guizhou, China

**Keywords:** freshwater fungi, macrofungi, marine fungi, morphology, phylogeny

Fungi, including both macro- and micro-ones, play a crucial role as natural bio-resources. There are an estimated 2.2–3.8 million fungal species worldwide, with more than 92% of them still unknown (Hawksworth and Lücking, 2017; Antonelli et al., 2020). Ascomycetes and Basidiomycetes with the largest number of species are the two main phyla of fungi (Bhunjun et al., 2022). Many fungal species in these two phyla provide important genetic and metabolite resources for our industry, agriculture, medicine, as well as ecological equilibrium. However, the knowledge of both phyla remains significantly missing and awaiting further research. Some of them are similar in morphology but have a far phylogenetic relationship, while others are closely related but have great morphological differences (Lu et al., 2018, 2022). Understanding the diversity and taxonomy of fungi in the Ascomycetes and Basidiomycetes are essential for unraveling their ecological roles, evolutionary relationships, and potential applications. In this Research Topic, we present a collection of research articles that delve into the exploration of fungal species, shedding light on their diversity, phylogeny, and morphological characteristics.

The articles in this issue span various fungal taxa and geographic regions, providing valuable insights into their classification and evolutionary history. The discoveries of new species within genera such as *Antrodia, Cantharellus, Thelephora, Steccherinum*, and *Podoscypha* highlight the hidden diversity within these groups. Through a combination of morphological observations and molecular phylogenetic analyses, these studies provide a comprehensive understanding of the relationships and unique characteristics of these newly described species.

Wang Y.-R. et al. focus on the genus *Megasporoporia sensu lato*, which has been extensively studied in China and South America. Through phylogenetic analyses, four independent clades representing four genera within *Megasporoporia sensu lato* have been identified. Building upon previous research, this study expands the sampling to tropical and subtropical regions in Asia, Oceania and East Africa. The phylogeny based on a comprehensive 4-gene dataset confirms the presence of four genera, viz *Jorgewrightia, Mariorajchenbergia, Megasporia* and *Megasporoporia sensu stricto*. The discovery of six new species and proposed combinations further enriches our understanding of this genus. The research provides a valuable identification key to the 36 accepted species, facilitating future studies on *Megasporoporia sensu lato*.

Song et al. focus on the genus *Hydnellum*, a group of ectomycorrhizal fungi with both ecological and medicinal significance. The species diversity of *Hydnellum* in China remains unclear, and this study aims to fill this knowledge gap. Through morphological characterization and phylogenetic analyses, five new species of *Hydnellum* are described, providing comprehensive descriptions, illustrations and phylogenetic trees. These findings contribute to our understanding of the diversity and evolutionary relationships within this important genus.

Wang Y. et al. explore the diverse family Boletaceae, which has attracted the attention of mycologists worldwide due to its morphological diversity and complex evolutionary history. Despite previous research, novel taxa continue to be described. This study introduces three new taxa and one new record from China, providing detailed morphological descriptions, color photographs and phylogenetic trees. These findings enhance our knowledge of the Boletaceae family and its evolutionary relationships.

Shifting our focus to the genus *Fistulina*, Zhou M. et al. discuss phylogenetic and morphological analyses of *Fistulina* samples from East Asia and North America. The study reveals the presence of two new species, *Fistulina americana* and *F. orientalis*, previously known as *F. hepatica*. Detailed descriptions of these species, including their distinguishing characteristics and habitats, are provided. This research expands our understanding of the diversity within the *Fistulina* genus.

The genus *Sanghuangporus*, known for its edible and medicinal properties, is explored in this Research Topic. Chen et al. model the current geographic distribution and predict future range shifts under climate change scenarios using occurrence records and MaxEnt modeling. The results provide valuable insights into the potential distribution of *Sanghuangporus* and its critical environmental variables, aiding in its utilization and conservation as an important edible and medicinal fungus.

Zhou H.-M. et al. focus on the genus *Antrodia*, with the description of two new species, viz *A. aridula* and *A. variispora*, from western China. Phylogenetic analyses based on a comprehensive six-gene dataset reveal that these species form two distinct lineages within the *Antrodia* genus. Morphologically different from existing *Antrodia* species, *A. aridula* is characterized by its annual and resupinate basidiocarps with angular to irregular pores, while *A. variispora* is characterized by its annual and resupinate basidiocarps with sinuous or dentate pores. These findings contribute to our understanding of the diversity and morphology of *Antrodia* species.

Li Y. et al. explore the diversity, taxonomy and phylogeny of five corticioid genera of Phanerochaetaceae in East Asia. Via morphological and molecular methods, seven new species are discovered, along with two suggested combinations and a proposed new name. The phylogenetic analyses reveal new lineages within the studied genera and provide insights into their evolutionary relationships. Detailed descriptions, illustrations and identification keys enhance our understanding of the species diversity within these corticioid genera. In another study, Li and He discover two new brown-rot corticioid fungi, viz *Coniophora beijingensis* and *Veluticeps subfasciculata*, in China. Through phylogenetic analyses, these species are identified and described based on their morphological characteristics. The findings contribute to our understanding of the diversity and taxonomy of wood-inhabiting fungi.

Zhang et al. focus on the genus *Cantharellus*, which is ecologically and economically important. Via morphological and molecular phylogenetic analyses, new species are described, including *C. bellus, C. cineraceus* and *C. laevigatus*, along with the previously described taxon *C. hygrophoroides*. The findings contribute to the taxonomic knowledge of *Cantharellus* subgenera *Afrocantharellus* and *Magni*, providing insights into their diversity and evolutionary relationships.

Furthermore, the genus *Thelephora*, a cosmopolitan group of ectomycorrhizal fungi, is investigated by Yang et al. via phylogenetic analyses and morphological observations, four new species are identified, namely *Th. aquila, Th. glaucoflora, Th. nebula* and *Th. pseudoganbajun*. These findings contribute to our understanding of the diversity and phylogenetic placement of *Thelephora* species in subtropical China.

Liu et al. explore the classification of Polyporales, a diverse group of wood-decaying fungi. Based on morphological characters and molecular data, two new families, viz Climacocystaceae and Gloeoporellaceae, are proposed within Polyporales. The phylogenetic analyses provide insights into the evolutionary relationships and divergence times within Polyporales. These new family classifications enhance our understanding of this diverse order.

The Research Topic also emphasizes the significance of employing both morphological and molecular approaches in studying fungal diversity. Phylogenetic analyses based on multiple gene sequences, including ITS, nLSU, mtSSU, TEF1, and RPB2, provide robust insights into the evolutionary relationships among different fungal lineages. These analyses are crucial for resolving taxonomic uncertainties and improving our understanding of fungal evolution.

Chin et al. focus on marine fungi, specifically those associated with sponges, algae and brown algae in Mauritius. Traditional methods of studying fungal diversity, such as culturing, have limitations in capturing the full spectrum of fungal communities. To overcome these challenges, high-throughput sequencing methods, specifically Illumina sequencing, were employed. The results provide valuable insights into the diversity and distribution of marine fungi in Mauritius, highlighting the significance of these organisms in nutrient cycling, organic matter decomposition, and symbiotic interactions.

Moving to northwestern Yunnan, China, Li L.-L. et al. explore the diversity of lignicolous freshwater fungi. Through meticulous studies over several years, new genera and species have been discovered, expanding our knowledge of these unique fungi. The research also emphasizes the importance of molecular phylogenetic analysis in establishing new taxa and understanding their relationships within the fungal kingdom.

Continuing the exploration of fungal diversity, Ma et al. and Li Q.-R. et al. investigate diatrypaceous fungi from southeastern Tibet and southern China. The integration of morphological features and molecular evidence reveals new genera and species, contributing to our understanding of the evolutionary relationships within Diatrypaceae. These findings highlight the richness of diatrypaceous fungi in China and their potential ecological significance.

Shifting the focus to wildlife, Hađina et al. examine the genotypes of *Malassezia pachydermatis*, a pathogenic yeast found in brown bears. The study reveals the presence of distinct genotypes, including one specifically adapted to brown bears. This research expands our understanding of the genotypic diversity of *M. pachydermatis* in the wild animal and highlights the potential for cross-species transmission.

Dong et al. explore the fungal community variation in phytoremediation, specifically in the context of quartz tailings soil. Phytoremediation, a sustainable and cost-effective approach, utilizes plants and fungi to remediate contaminated soil. The study demonstrates the positive impact of plant-fungi-urban sludge combinations on fungal diversity and soil properties, providing valuable insights for future phytoremediation projects.

In conclusion, this Research Topic highlights the importance of exploring and understanding the diversity and taxonomy of fungal species. The research presented here expands our knowledge of these species, their evolutionary relationships, and ecological significance. We hope that this Research Topic of articles will inspire further exploration and research, ultimately contributing to a comprehensive understanding of the remarkable world of fungi and their ecological significance.

## Author contributions

Y-ZL wrote the original draft. J-KL, JC, X-YZ, and J-CK revised the article. All authors agreed to the published version of the article.
